# Assessing the Heterogeneity of the Fc-Glycan of a Therapeutic Antibody Using an engineered FcγReceptor IIIa-Immobilized Column

**DOI:** 10.1038/s41598-018-22199-8

**Published:** 2018-03-02

**Authors:** Masato Kiyoshi, Jose M. M. Caaveiro, Minoru Tada, Hiroko Tamura, Toru Tanaka, Yosuke Terao, Koldo Morante, Akira Harazono, Noritaka Hashii, Hiroko Shibata, Daisuke Kuroda, Satoru Nagatoishi, Seigo Oe, Teruhiko Ide, Kouhei Tsumoto, Akiko Ishii-Watabe

**Affiliations:** 1 Division of Biological Chemistry and Biologicals, National Institute of Health Sciences, Tokyo, 158–8501 Japan; 2 Department of Bioengineering, School of Engineering, The University of Tokyo, Tokyo, 113–8656 Japan; 3 Graduate school of Pharmaceutical Sciences, Kyushu University, Maidashi, Higashi-ku, Fukuoka, 812–8582 Japan; 4 Tosoh Corporation, Hayakawa, Ayase, Kanagawa 252–1123 Japan; 5 Institute of Medical Sciences, The University of Tokyo, Shirokanedai, Minato-ku, Tokyo, 108–8639 Japan; 6 Laboratory of Pharmacokinetic Optimization, Center for Drug Design Research, National Institutes of Biomedical Innovation, Health and Nutrition, Ibaraki City, Osaka, 567–0085 Japan

## Abstract

The N-glycan moiety of IgG-Fc has a significant impact on multifaceted properties of antibodies such as in their effector function, structure, and stability. Numerous studies have been devoted to understanding its biological effect since the exact composition of the Fc N-glycan modulates the magnitude of effector functions such as the antibody-dependent cell mediated cytotoxicity (ADCC), and the complement-dependent cytotoxicity (CDC). To date, systematic analyses of the properties and influence of glycan variants have been of great interest. Understanding the principles on how N-glycosylation modulates those properties is important for the molecular design, manufacturing, process optimization, and quality control of therapeutic antibodies. In this study, we have separated a model therapeutic antibody into three fractions according to the composition of the N-glycan by using a novel FcγRIIIa chromatography column. Notably, Fc galactosylation was a major factor influencing the affinity of IgG-Fc to the FcγRIIIa immobilized on the column. Each antibody fraction was employed for structural, biological, and physicochemical analysis, illustrating the mechanism by which galactose modulates the affinity to FcγRIIIa. In addition, we discuss the benefits of the FcγRIIIa chromatography column to assess the heterogeneity of the N-glycan.

## Introduction

The importance of IgG therapeutic antibodies as a tool for combating several diseases has greatly expanded over the past several decades. More than 40 antibodies and derivatives have been approved for the treatment not only of cancer, but in recent times also for various other diseases, such as atopic dermatitis (Dupilumab in 2015 in the US)^1^, familial hypercholesterolaemia (Alirocumab in 2015 in the US)^2^, or serious bleeding of patients taking Dabigatran (Idarucizumab in 2015 in the US)^3^. In the realm of cancer treatment, novel therapeutic antibodies against immune checkpoints have been developed by pharmaceutical industries, opening the possibility to eradicate cancer cells by inhibiting these crucial molecular switches^4^. In addition, the dramatic progress of bioengineering techniques is fostering vigorous research to generate antibody drugs with tailored properties and improved efficacy, such as for example bispecific and glyco-engineered antibodies, and antibody-drug-conjugates (ADC). These approaches are undoubtedly strengthening the benefits of therapeutic antibodies to treat a wide range of human diseases with increased advantages for patients and the medical community.

Therapeutic antibodies expressed in Chinese hamster ovary (CHO) and other mammalian cells display a molecule of carbohydrate (N-glycan) attached to residue Asn297 of the fragment crystallizable (Fc) region^5^. Such N-glycan comprises a constant core region composed of two *N*-acetyl-glucosamine (GlcNAc) and three mannose (Man) units. The core region is generally enlarged with additional saccharides of fucose (Fuc), GlcNAc, galactose (Gal), and sialic acids (*N*-acetylneuraminic acid or *N*-glycolylneuraminic acid). Because these saccharides appear in variable compositions, the oligosaccharide attached to Asn297 exhibits significant heterogeneity^6,7^. The glycosylation of each of the two chains of Fc further increases the diversity of the glycosylation pattern, resulting in heterogeneity also at the functional level. The influence of glycosylation is demonstrated by its modulation of key effector functions such as the complement-dependent cytotoxicity (CDC) and the antibody-dependent cell-mediated cytotoxicity (ADCC)^7,8^.

CDC is a cytotoxic effect initiated by binding of C1q to the Fc region of antibody. Subsequently, the complement components C2 to C9 induce clustering, starting the classical complement cascade. Ultimately the complement cascade induces the formation of the membrane attack complex (MAC) eliciting cell lysis^9^. In this sense, the composition of the N-glycan of Fc and in particular the presence of a terminal galactose enhances the intensity of the CDC response^10,11^.

ADCC is a cytotoxic effect elicited by immune cells, such as natural killer (NK) cells and macrophages. Fcγ receptor IIIa (FcγRIIIa) governs this response by engaging immune complexes (IC) to these immune cells. Upon binding of IC to FcγRIIIa, the immune cells are activated, leading to the release of cytotoxic molecules such as perforin and granzyme. The correlation between the intensity of ADCC and the glycosylation of Fc has been studied intensively, showing that the removal of fucose significantly increases the effect of ADCC activity^12–14^. More importantly, the preparation of afucosylated antibodies resulted in an effective strategy to improve their therapeutic capabilities as demonstrated by the approval of two glyco-engineered antibodies for the treatment of human diseases^15,16^.

There is a strong interest in reaching a comprehensive and predictable understanding of the effect of glycosylation on antibody properties^17–21^ In particular, the fundamental principles explaining the influence of the galactose units of the N-glycan in the physicochemical properties of the interaction between IgG-Fc and FcγRIIIa is critical for the molecular design, manufacturing process optimization, and quality control of therapeutic antibodies.

Herein we have addressed the mechanism by which the terminal units of galactose of the N-glycan of IgG-Fc modulate its affinity for FcγRIIIa. We employed a chromatographic column in which an engineered FcγRIIIa was immobilized. We demonstrate that the antibodies studied are separated according to their level of galactosylation, and determine their biological, structural, and physicochemical properties. On the basis of those analyses, we have discussed the recognition mechanism between of IgG-Fc and FcγRIIIa. We concluded that the CH2 dynamics, which is affected by the composition of the N-glycan, is an important factor to be taken into account when developing therapeutic antibodies, especially in terms of their effector function, and overall stability, which are readily analyzed by a novel FcγRIIIa-immobilized column.

## Materials and Methods

### Protein cloning, expression and purification of FcγRIIIa muteins

The amino acid sequence of mutated residues is shown in Supplemental Fig. 1. The DNA encoding Mut FcγRIIIa was expressed in vector pTrc99a (GE Healthcare) displaying a hexa-histidine tag at the C-terminus. Cells of *Escherichia coli* strain BL21 (DE3) (Novagen) transformed with expression vector were grown at 37 °C in 2 × YT broth. Isopropyl β-D-1-thiogalactopyranoside was added (0.01 mM) when the optical density at 600 nm reached 0.6. The culture broth was harvested 16 h at 20 °C after induction. The cells were harvested by centrifugation at 8,000 × g for 10 min and the pellet thus obtained was resuspended in a solution containing 0.5 M NaCl and 50 mM Tris-HCl at pH 8.0 (buffer A). Cells were subsequently lysed by the sonication method with a disruptor instrument (Tommy) for 15 min. A pellet containing the soluble intracellular components was obtained by centrifugation at 40,000 × g for 30 min. The soluble fraction was collected and applied onto a Ni-NTA column (Novagen) equilibrated with buffer A. Protein was eluted with 300 mM imidazole contained in buffer A. Mut FcγRIIIa containing fractions were further purified by size exclusion chromatography using a HiLoad 26/60 Superdex 75-pg column (GE Healthcare) equilibrated with 50 mM TrisHCl, 200 mM NaCl at pH 7.4. Mut FcγRIIIa showed over 99% purity as judged by SDS-PAGE analysis (data not shown).

WT FcγRIIIa expressed by HEK293 cells was obtained from Sino Biologicals (Catalog #10389-H08C1-50). The purity of WT FcγRIIIa was of over 95% as determined by SDS-PAGE. The glycosylation pattern was not reported by the vendor.

### Chromatography

The affinity column in which an optimized version (Supplemental Fig. 1) of FcγRIIIa was immobilized to a solid support (4.6 mm I.D. × 75 mm) was from TOSOH (Tokyo, Japan). We used non-glycosylated FcγRIIIa as the ligand of the column because it would be very difficult to ensure the quality of column if heterogeneously glycosylated receptor was immobilized. Mut FcγRIIIa was immobilized on a non-porous methacrylate resin (5 μm) by optimized highly orientated coupling method on residues Lys, Cys, Asp and Glu of Mut FcγRIIIa. Mobile phase A was 20 mM sodium acetate, 50 mM NaCl at pH 4.5, and mobile phase B was 10 mM glycine hydro-chloride at pH 3.0. A total of 1 mg of Rituximab (Roche) was diluted 50-fold with mobile phase A to adjust the pH to 4.5. A linear gradient of buffer B (0 to 100%) at a flow rate of 0.5 mL/min was applied to the column for 40 min to elute the antibody employing an Äkta purifier system (GE Healthcare).

### Surface plasmon resonance (SPR)

The interaction between WT (or Mut) FcγRIIIa and Rituximab was analyzed by SPR in a Biacore T200 instrument (GE Healthcare). Proteins were dialyzed against running buffer (PBS supplemented with 0.005% Tween-20). A NTA sensor chip (GE Healthcare) was used for immobilization of FcγRIIIa. Before each run, the NTA sensor chip was treated with 500 mM nickel chloride at 10 μL/min for 1 min to load the sensor chip with Ni^2+^ ions. Subsequently, FcγRIIIa was immobilized on the surface chip at a density of 350 RU. Sensorgrams corresponding to the binding of Rituximab to FcγRIIIa were obtained by injecting increasing concentrations of analyte at a flow rate of 30 μL/min. Contact and dissociation time were 150 s and 250 s, respectively. Regeneration was carried after completion of each sensorgram by injecting a solution of 0.35 M EDTA. Data analysis was performed with the BIAevaluation software (GE Healthcare). Association (*k*_on_) and dissociation (*k*_off_) rate constants were calculated by a global fitting analysis assuming a Langmuir binding model and a stoichiometry of (1:1). The dissociation constant (*K*_D_) was determined from the ratio of the kinetic rate constants as follows:

1 $${K}_{{\rm{D}}}={k}_{{\rm{off}}}/{k}_{{\rm{on}}}$$

### Thermodynamic analysis

SPR analysis was performed as described above. The changes in enthalpy and entropy were calculated as previously reported^22^. Changes in enthalpy (Δ*H°*) and entropy (Δ*S°*) were calculated from the slope and intercept, respectively, of the temperature dependence of the dissociation constant using the van’t Hoff approximation:

2 $$\mathrm{ln}\,{K}_{{\rm{D}}}={\rm{\Delta }}H^\circ /RT+{\rm{\Delta }}S^\circ /R$$

where *R* is the gas constant and *T* is the absolute temperature.

### Glycan analysis

The analytical method to determine the composition of the N-gycan comprised three steps: Release of N-linked oligosaccharides, labeling with 2-aminobenzamide (2-AB), and UPLC.

The N-linked oligosaccharides were released enzymatically using PNGase F (Roche). 20 μg of antibody were mixed with 1 U of PNGase F. The mixture was incubated at 37 °C for 16 h, and the resulting digested compounds loaded onto an Envi-Carb graphitized carbon column (Spelco, USA) pre-treated with 1.0 mL acetonitrile. After a washing step with 3.0 mL water, the N-glycan was eluted with 1.0 mL of 5 mM ammonium acetate in 50% acetonitrile. The eluted fractions were collected and dried with a centrifugal evaporator (Speedvac, Thermofisher).

The labeling solution (10 μL of 0.37 M 2-AB and 1 M NaCNBH3 in 70:30 (v:v) DMSO:acetic acid) was added to dried samples of N-glycan. The reaction mixtures were incubated at 65 °C for 3 h. Acetonitrile (1 mL) was gently added to the labeled glycan sample solution, followed by centrifugation at 15,000 × g for 10 min. The supernatant was discarded, and the pellet containing the labeled glycan was dissolved in 20 μL of distilled water. A total of 1 μL of glycan solution was subjected to a Waters ACQUITY UPLC BEH amide column (2.1 × 150 mm, 1.7 μm) in a Waters ACQUITY UPLC H-class system. Mobile phase A was 0.1 M ammonium formate pH 4.6, and mobile phase B was 100% (v/v) acetonitrile. A linear gradient (A; 25% B; 75% to A; 50% B; 50%) for 50 min was applied and fluorescence signals were detected at 420 nm (Excited at 330 nm). The structure of the glycans of each peak was determined based on elution profile^23^.

### FcγRIIIa activation assay (corresponds to ADCC)

We established a Jurkat cell line expressing WT FcγRIIIa (Jurkat/FcγRIIIa/NFAT-Luc) for assessing the receptor activation via NFAT-driven expression of luciferase gene^24^. Daudi (JCRB9071) cells expressing CD20 were obtained from the JCRB Cell Bank and cultured in RPMI 1640 medium supplemented with 20% FBS. Daudi cells (1 × 10^4^ cells/well) and Jurkat/FcγRIIIa/NFAT-Luc cells (5 × 10^4^ cells/well) suspended with Opti-MEM I Reduced Serum Media (Thermo Fisher Scientific) were seeded in a 96-well plate with serially diluted Rituximab. After incubation for 5 h at 37 °C in 5% CO_2_, the luciferase activities were determined by using the ONE-Glo Luciferase Assay System (Promega) in an EnSpire Multimode Plate Reader (PerkinElmer). The measurements were performed in triplicate.

### CDC

Daudi cells (1 × 10^4^ cells/well) suspended with Opti-MEM I Reduced Serum Media were seeded in a 96-well plate with serially diluted Rituximab. A final concentration of 16% (v/v) human serum was added as a source of complement. After incubation for 2 h at 37 °C in 5% CO_2_, cell lysis was measured with a CytoTox-Glo^TM^ Cytotoxicity Assay kit (Promega) in an EnSpire Multimode Plate Reader (PerkinElmer). The measurements were performed in triplicate.

### CD20-binding assay

After washing twice with buffer (PBS supplemented with 0.5% bovine serum albumin, 2 mM Na-EDTA and 0.05% sodium azide), the Daudi cells were suspended in the same buffer containing serially diluted antibody and incubated on ice for 30 min. After that, the cells were further incubated with Alexa488-conjugated F(ab’)_2_ anti-human IgG Fc (Jackson ImmunoResearch) on ice for another 30 min. After washing twice with the buffer, the cells were analyzed by using a FACSCanto II flow cytometer (BD Biosciences, San Diego, CA). The measurements were performed in triplicate.

### Crystallization

The Fc fragment of Rituximab (Roche) was obtained by papain digestion and purified with a Protein A kit (Pierce) following the instructions of the manufacturer. Purified IgG-Fc was mixed with purified Mut FcγRIIIa (Supplemental Fig. 1) at a molar ratio of 1:1, and the complex subjected to size exclusion chromatography using a HiLoad 26/60 Superdex 200-pg column (GE Healthcare) equilibrated with 20 mM Tris-HCl, 100 mM NaCl at pH 7.4. The fractions containing the IgG-Fc in complex with Mut FcγRIIIa were concentrated to 5.0 mg/mL. A crystallization screening was carried out using an Oryx8 instrument (Douglas Instruments). After several rounds of optimization, single crystals were obtained in a solution of 14% PEG 3,350, 100 mM NaCl.

### X-ray data collection and structure determination

Suitable crystals were harvested, cryoprotected in mother liquor supplemented with 25% glycerol, and stored in liquid N_2_. Data collection was carried out at beamline BL5A of the Photon Factory (Tsukuba, Japan) at 100 K. Diffraction images were processed with MOSFLM^25^. Diffraction data were merged and scaled with SCALA^26^. The structure was determined by the method of molecular replacement with PHASER^27^ using the coordinates of the same complex with glycosylated receptor (PDB entry code 3AY4)^28^. The crystallographic models were refined with REFMAC^29^. Manual building was carried out with COOT^30^. Validation of the refined structure was performed with COOT and PROCHECK^31^. No outliers in the Ramachandran plot were observed. The coordinates of the complex have been deposited in the PDB with accession code (5YC5).

### Hydrogen/Deuterium exchange mass spectrometry (HDX MS)

Rituximab at 2 mg/mL was diluted 20-fold with phosphate buffer saline in D_2_O. The diluted solutions were separately incubated at 10 °C for 0, 0.17, 1, 10, 60, and 240 min. Deuterium-labeled samples were quenched by adjusting the pH to 2.5 with equal volumes of pre-chilled quenching buffer (4 °C) containing 300 mM Tris (2-carboxyethyl) phosphine hydrochloride (TCEP-HCl, Sigma-Aldrich) and 4 M guanidine-HCl (Thermo Fisher Scientific, CA, USA) using the CTC PAL sample manager (LEAP Technologies, NC). All time points were determined with three independent labeling experiments.

After quenching, the solutions were subjected to online pepsin digestion and analyzed by LC/MS using the nanoACQUITY UPLC system (Waters) connected to the SynaptG2-S Q-TOF mass spectrometer (Waters). Online pepsin digestion was performed using a Poroszyme Immobilized Pepsin Cartridge (2.1 × 30 mm, Applied Biosystems, CA, USA) in formic acid solution (pH 2.5) for 2 min at 10 °C at a flow rate of 300 μL/min. As analytical column, a reversed-phase ACQUITY UPLC BEH C18 column (1.0 × 100 mm, 1.7 mm, Waters) was used. The mobile phase was 0.1% formic acid solution (A buffer) and 0.1% formic acid containing 90% acetonitrile (B buffer). The deuterated peptides were eluted at a flow rate of 30 μL/min with a gradient of 13% to 85% of B buffer in 8 min. The conditions of the mass spectrometer were as follows: electrospray voltage of 3.0 kV, positive ion mode, trap collision energy at 4.0 V, sampling cone at 40 V, source temperature at 80 °C, desolation temperature at 100 °C, and mass range of *m/z* 100 to 2,000. MS^E^ was performed by a series of low–high collision energies ramping from 10 to 40 V. The MS^E^ spectra were subjected to a database search analysis using the ProteinLynx Global Server (PLGS) 2.5.3 against an in-house database containing the amino acid sequence of Rituximab. The search results and MS raw files were used for the analysis of the deuteration levels of the peptic fragments using the DynamX 2.0 software (Waters). The measurement was repeated 3 times.

### Differential scanning calorimetry (DSC)

Thermal stability of each IgG glycovariant, Fab, Fc, and complete IgG (Rituximab) was determined using differential scanning calorimetry (DSC) in a VP-DSC instrument (Malvern, UK). Protein samples were dialyzed against PBS prior to each scan. Protein sample at a concentration of 10 μM was subjected to a temperature ramp between 10 and 100 °C at a scan rate of 1.0 °C/min. The thermogram of the protein sample was normalized by subtracting the signal form the reference cell containing only buffer. The melting temperature (*T*_*M*_) values were calculated by a standard fitting procedure using Microcal Origin 7.0 software and a non-two state model.

## Results

### Antibody separation based on N-terminal glycan using Mut FcγRIIIa-immobilized column chromatography

**Figure 1:**
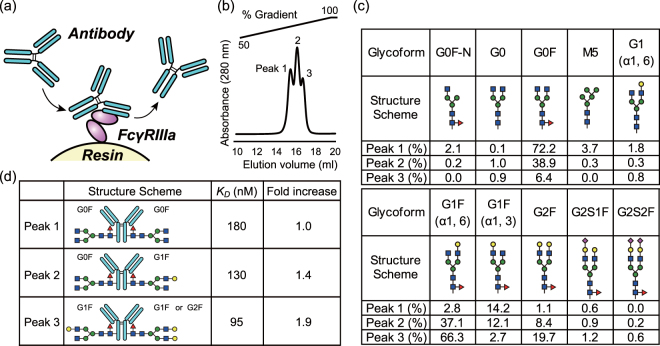
Glycoforms present in the fractions of antibody separated by FcγRIIIa-immobilized column chromatography. (**a**) Schematic representation of the chromatographic column. The antibody and the molecule of FcγRIIIa are colored in cyan, and purple, respectively. (**b**) Chromatographic profile of Rituximab. (**c**) Major glycoforms present in antibody separated from above (peaks 1–3). Blue squares, green circles, yellow circles, magenta diamonds, and red triangles correspond to GlcNAc, Man, Gal, sialic acid, and Fuc, respectively. (**d**) Schematic representation of the glycoform predominately observed in each peak. The absolute and relative affinities for the WT FcγRIIIa (glycosylated) as determined by SPR are also shown.

Recently, a separation technology to purify IgG from complex mixtures employing FcγRIIIa-immobilized chromatography has been developed by TOSOH (Fig. 1a). A key development in this technology is the preparation of stable recombinant FcγRIIIa suitable for expression in *E*. *coli*, rendering a non-glycosylated receptor. FcγRIIIa was mutated for increasing stability in the absence of proper glycosylation, and to resist the harsh environment of the purification column such as low and high pH, and the constant shearing flow of solutions. The purified Mut FcγRIIIa protein is immobilized on the surface of the TSK-gel (TOSOH) resin.

Rituximab was loaded onto the column and eluted by linear pH gradient from 4.5 to 3.0. The antibody was eluted according to the affinity to immobilized Mut FcγRIIIa. The elution profile of injected antibody showed three partially overlapping peaks between 40 and 80% buffer B (Fig. 1b). One possible explanation for the separation profile could be the existence of glycosylation variants that could influence the binding affinity to the immobilized receptor. The collected peaks were thus conserved for further analysis (see below). The Mut FcγRIIIa-immobilized column was also employed for analytical purposes with other IgG molecules including mutants intended to enhance or reduce the biological activity (ADCC, and/or CDC, and/or phagocytosis) resulting in various retention profiles (Supplemental Fig. 2)^32–39^. The variant e3 (mutated to abrogate Fc binding) does not bind to the column, while Mogamulizumab (afucosylated), variants e5 and e9 (mutated to increase the affinity to FcγRIIIa) showed late retention times. Thus, the retention time provides first-hand information that helps to assess the affinity to FcγRIIIa (corresponding ADCC). Three Rituximab (Roche, In-house, India) showed slightly different elution profiles, such as retention time, width, and height. The mutants engineered to strengthen the affinity to FcγRIIIa (e5, e9) exhibited less well separated peaks, probably because of that the elution method was not specifically optimized for these mutants.

### Glycan analysis

To elucidate the N-glycosylation profiles of peaks 1–3 from above, we employed a procedure consisting in hydrolysis of the N-glycan, followed by labeling with the dye 2-AB, and separation of the different species with hydrophilic interaction liquid chromatography ultra-high performance liquid chromatography (HILIC-UPLC). This procedure revealed 10 major species, all of them typically found in the IgG-Fc region of therapeutic antibodies (Fig. 1c). The main difference between peak 1, 2, and 3 was the increasing content of galactose, which correlated with the retention time in the column. Peak 1 contained N-glycan of the form G0F as the main component (72.2%), whereas in peak 2 approximately equal amounts of G0F (38.9%) and G1F (α1, 6) (37.1%) were found. Peak 3 contained a major fraction of G1F (α1, 6) (66.3%) and a minor fraction of G2F (19.7%). In all cases, the afucosylated glycan was present as a minor component (<4%). A small percentage of high mannose glycan (M5) appeared in peak 1, representing 3.7% of the total carbohydrate in that fraction. Interestingly, the ratio of arm galactosylation (G1F (α1, 6)/G1F (α1, 3)) increased dramatically at longer retention times. Specifically, the relative values increased by more than 100-fold, from 0.19 in peak 1, to 3.1 in peak 2, and 24.6 in peak 3.

### Binding affinity of IgG to FcγRIIIa

Since glycosylation may affect the binding affinity to the receptor^5^, we hypothesized that the order of elution reflected differences in affinity. To quantify the differences of affinity between each peak of IgG1 and FcγRIIIa in a quantitative fashion we employed SPR. For this experiment, both the optimized non-glycosylated as well as the WT glycosylated receptor were monitored in parallel. A Ni-NTA sensor chip decorated with receptor was employed as stationary phase, and the kinetic characterization was evaluated from sensorgrams of each IgG glycovariant injected as an analyte onto the sensor chip. The kinetic parameters obtained are given in Supplemental Table 1, whereas the values of the equilibrium constant (*K*_*D*_) for the WT receptor are shown in Fig. 1d. As expected from the separation step, the strength of the binding correlated with the position in the chromatographic step in the order peak 1 < peak 2 < peak 3 for both the WT glycosylated and the optimized non-glycosylated receptors. The determined values of *K*_*D*_ indicated that the binding affinities of peaks 2 and 3 with respect to peak 1 increased 1.4-fold and 1.9-fold, respectively. These differences are largely associated to a decrease in the dissociation rate constant (*k*_*off*_) and not to the association rate constant (*k*_*on*_) which remains essentially unchanged (SI Table 1).

A very similar trend was observed for the optimized non-glycosylated receptor (Supplementary Table S1), although we note that the absolute affinity is about 8-fold lower than that of the WT-glycosylated receptor. This is consistent with the decrease of affinity (6~8 fold) in a variant of the receptor not glycosylated at the critical Asn162 position (mutated to Gln) using SPR^40^.

### Effector functions

**Figure 2:**
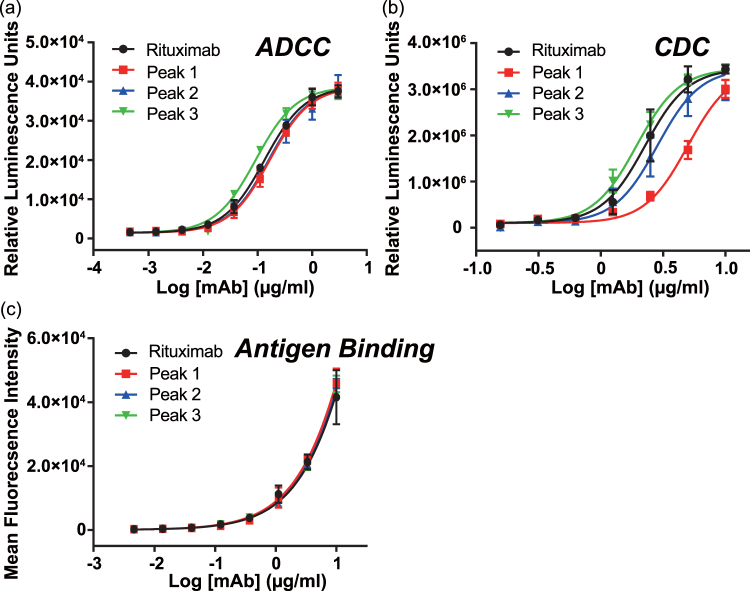
Effector function of the purified fractions. (**a**) Jurkat cell activation through engagement with immune-complex via FcγRIIIa (corresponds to ADCC) was evaluated by luciferase activity. (**b**) CDC was assessed by luciferase activity in the presence of human serum. (**c**) The binding level of antibody to the antigen-presenting cell was measured by flow cytometry using Alexa488-conjugated F(ab’)_2_ anti-human IgG-Fc.

To determine the ADCC activity of antibody, we employed a WT FcγRIIIa-expressing cell-based reporter assay of high sensitivity established by Tada *et al*.^24^. The relative potency of each glycoform was calculated from the value of EC_50_ (Fig. 2a, Table 1). The sample corresponding to peak 3 showed the stronger FcγRIIIa activation ability (1.5-fold increase stronger than the parent antibody), while the antibody from peak 1, and 2 showed weaker activations (80% of the reference level). These insights demonstrate a positive correlation between the galactose content of the N-glycan and the ADCC activity, consistent with the study of Thomann *et al*.^41,42^.

**Table 1:** Biological potency.

ADCC		
Protein	EC_50_ (μg/mL)	Relative potency (%), 95% confidence interval
Rituximab	0.13	100
Peak 1	0.20	80 (70.4~92.9)
Peak 2	0.19	84 (74.0~97.6)
Peak 3	0.05	146 (128.0~168.0)
**CDC**		
	**EC**_**50**_ (**μg/mL**)	**Relative Potency** (**%**), **95% confidence interval**
Rituximab	2.3	100
Peak 1	5.0	45 (40.3~52.0)
Peak 2	2.8	80 (70.8~91.2)
Peak 3	1.9	117 (104.0~134.1)

We also examined the level of CDC response. The relative potency was also estimated from the determined values of EC_50_ (Fig. 2b, Table 1). The reference value of EC_50_ obtained for Rituximab before the separation step was 2.3 μg/mL. Among the three major glycovariants, the sample corresponding to peak 3 showed enhanced CDC activity (120% to that of the reference sample). In contrast, the fractions corresponding to less galactosylated antibody showed decreased CDC activity (45% and 80% for peak 1 and peak 2, respectively).

To evaluate the binding level of the antibody on the antigen-expressing cell surface, we employed FACS analysis. The binding level of antibody to antigen-presenting cell was determined from the fluorescence of anti-human Fab antibody F(ab’)_2_ labeled with Alexa488. As shown in Fig. 2c, the binding levels of samples corresponding to peak 1, 2 and 3 were indistinguishable from each other, discarding the possibility that the changes observed are related to the performance of the Fab region of the antibody. We concluded that the terminal galactose does not change the affinity of the antibody for the antigen.

### Crystal structure

**Figure 3:**
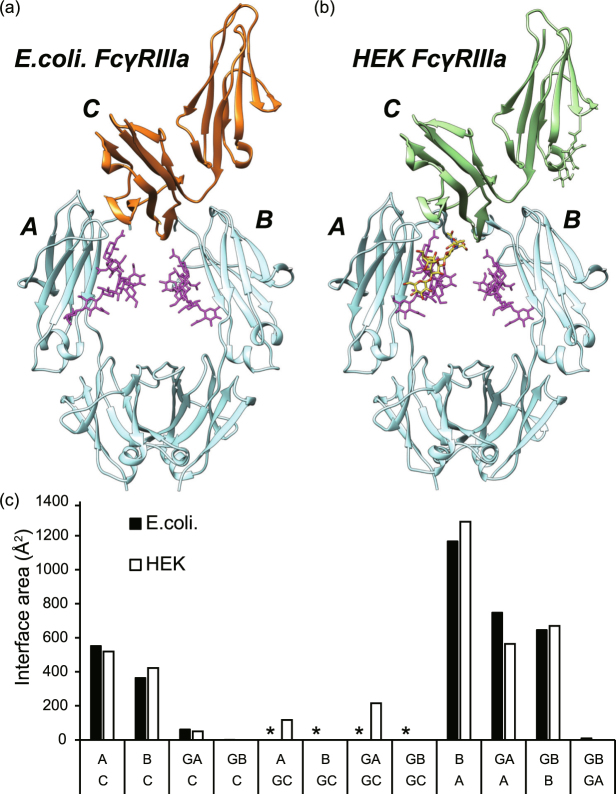
Comparison of structures of glycosylated and non-glycosylated FcγRIIIa in complex with IgG-Fc. (**a**) Crystal structure of Fc-FcγRIIIa complex. Recombinant Mut FcγRIIIa was expressed in *Escherichia coli* (non-glycosylated, left panel). (**b**) Previously reported crystal structure of Fc-FcγRIIIa complex (right panel, PDB ID: 3SGJ). Non-glycosylated FcγRIIIa, glycosylated FcγRIIIa, Fc, and the glycan attached to Fc are depicted in orange, light green, cyan, and magenta, respectively. The N-glycan molecule attached to Asn168 of FcγRIIIa is depicted in yellow sticks. The letters A, B, and C represent chains A and B of Fc, and FcγRIIIa, respectively. (**c**) Buried surface area (Å^2^) calculated by the PISA server^51^. GA, GB, GC indicate the N-glycan attached to Fc (chain A), Fc (chain B), and FcγRIIIa, respectively. The asterisks indicate that no interaction is possible for the non-glycosylated FcγRIIIa produced in *E*. *coli*.

Since the FcγRIIIa immobilized on the purification column (Supplemental Fig. 1) has been mutated and expressed as a recombinant protein in the non-glycosylated form, it was deemed necessary to verify the molecular basis of the recognition for IgG-Fc. For that purpose, we determined the crystal structure of the complex at a resolution of 2.7 Å. The crystallographic data and refinement statistics are shown in Supplemental Table 2. A comparison of the crystal structures of the complex with IgG-Fc of the non-glycosylated and glycosylated receptor is shown in Fig. 3a,b. From the visual comparison and the RMSD values obtained after superposition of the two crystal structures, no significant differences were observed. The RMSD of main chain atoms in chain A of Fc, chain B of Fc, and FcγRIIIa were 0.78 Å, 0.59 Å, and 1.1 Å, respectively. We also determined the RMSD with respect to other reported crystal structure (3SGK: 0.5 Å, 0.59 Å, 0.87 Å, 5XJE: 0.59 Å, 0.78 Å, 0.69 Å, 5XJF 0.62 Å, 0.82 Å, 0.61 Å, 5BW7: 0.66 Å, 0.83 Å, 0.60 Å, 3AY4: 0.64 Å, 0.82 Å, 0.68 Å, were for each case the RMSD corresponds to chain A, chain B, chain C, respectively)^28,43–45^. Consistent with previously reported crystals structures^28,43^, no direct contact of the terminal galactose of the N-glycan of IgG-Fc and Mut FcγRIIIa was observed. The contact interface between the protein and N-glycan portions of IgG-Fc and the receptor are summarized in Fig. 3c, again showing no significant differences between the two crystal structures.

### The glycosylation regulates the dynamics of Fc

**Figure 4:**
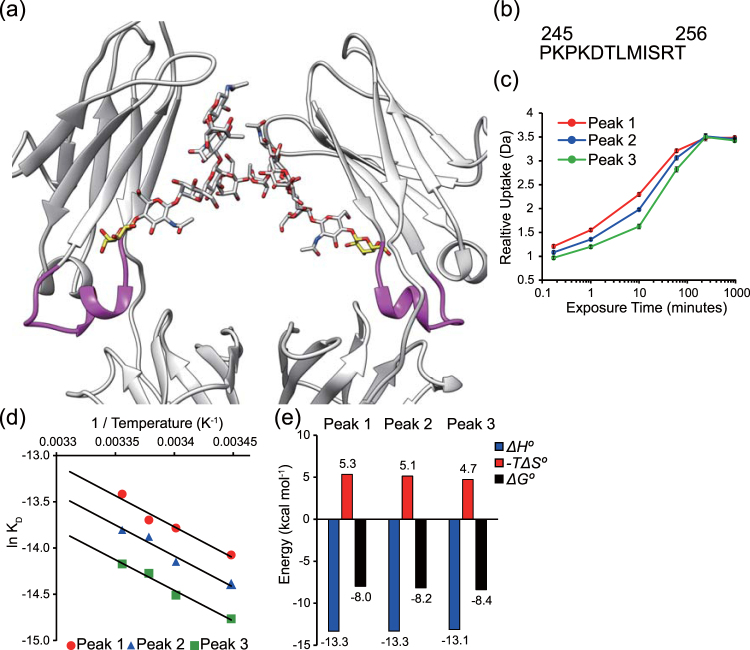
Dynamic and thermodynamic behavior of different glycoforms of IgG-Fc. (**a**) The peptide where each glycoform of IgG-Fc produced differences in deuterium uptake is depicted in magenta. The terminal galactose units are depicted with yellow sticks. (**b**) Sequence of the peptide of IgG-Fc showing differential deuterium exchange rates. (**c**) Relative deuterium uptake for each glycoform. (**d**) Representation of the van’t Hoff plot corresponding to the binding of each glycoform to a surface decorated with Mut FcγRIIIa. Temperature dependence of the dissociation constant *K*_D_ yields the van’t Hoff enthalpy (*ΔH°*), entropy (−*TΔS°*) and free energy (*ΔG°*). (**e**) Comparison of the values of the thermodynamic parameters determined from panel d.

The crystal structure did not show direct contacts of the galactose units of the saccharide with the receptor that could explain their effect on the affinity for FcγRIIIa. Based on the collective evidence gathered so far^5^, it has been proposed that the galactose moiety could influence the dynamic and conformational ensemble of IgG-Fc. We employed hydrogen-deuterium exchange mass spectrometry (HDX-MS) using the purified IgG glycovariants. The deuterium uptake increases in the peptide ranging from 245 P to 256 T is the order Peak 1 > Peak 2 > Peak 3, implicating that this particular peptide exhibits a more relaxed conformation as the fraction of galactose units decreases (Fig. 4a–c). The increased dynamic behavior of the peptide 245 P to 256 T is consistent with previously reported study by Houde *et al*.^46^. The peptide is located at a critical region between CH2 and CH3 domains and in the immediate environment of the galactose unit^5^ although in our crystal structure, the galactose moiety was not observed because of conformational disorder, a circumstance very often happening in other crystal structures of IgG-Fc.

We employed differential scanning calorimetry (DSC) analysis to determine the effect of galactosylation on the thermal stability, *i*.*e*. the melting temperature (*T*_*M*_) and denaturation enthalpy (*ΔH*). The results indicated that the antibody with the highest content in galactose, peak 3, exhibited the greatest denaturation enthalpy. In contrast, no appreciable differences in the value of *T*_*M*_ were found among the antibody fractions (Supplemental Fig. 3, Supplemental Table 3). These results suggest that the terminal galactose engages in noncovalent interaction with surrounding residues, consistent with the identity of the dynamic peptide identified above by HDX-MS.

To further assess the dynamic behavior of Fc, we determined thermodynamic parameters of the binding with Mut FcγRIIIa using SPR. Because these experiments require a long time and stable sample is needed, the optimized and more stable non-glycosylated FcγRIIIa was employed. The van’t Hoff change of enthalpy (*ΔH*) and the change of entropy (−*TΔS*, calculated at 25 °C)^47,48^ of the binding between Mut FcγRIIIa and the three IgG variants isolated (Peak 1, 2 and 3) were determined from the temperature dependence of the dissociation rate constant. In the three instances evaluated the antibody exhibited negative and favorable change of enthalpy (*ΔH*_Peak1_ = −13.3 kcal/mol, *ΔH*_Peak2_ = −13.3 kcal/mol, *ΔH*_Peak3_ = −13.1 kcal/mol), and was opposed by the change of entropy (−*TΔS*_Peak1_ = 5.3 kcal/mol, −*TΔS*_Peak2_ = 5.1 kcal/mol, and –*TΔS*_Peak3_ = 4.7 kcal/mol). These results suggested that the antibody binds to the receptor with favorable intermolecular interactions, and it is opposed by the unfavorable entropy loss (Fig. 4d,e). The value of entropy change decreased as the content of galactose increased, suggesting a reduction of the conformational entropy of the antibody when the N-glycan contained a greater fraction of galactose.

## Discussion

The glycosylation level of Fc has a significant impact on effector functions, including ADCC, and CDC. Especially, ADCC elicited by engagement of immune complex and FcγRIIIa, is a major function of therapeutic antibodies. Therefore, a detailed understanding of the correlation between glycosylation and affinity to FcγRIIIa is crucial for managing the effector function of therapeutic antibodies.

In this work, we separated antibody fractions as a function of their affinity to a FcγRIIIa-immobilized column. We obtained three peaks, reflecting an increasing affinity to the receptor and positively correlated with the abundance of terminal galactose in the glycan attached to Asn297.

Previous studies suggested that the affinity of fucosylated antibody to FcγRIIIa is reduced when compared to afucosylated antibodies since the fucose moiety clashes with the receptor glycan attached to Asn162^28,43^. However, the separation described herein using FcγRIIIa-immobilized column showed an elution peak corresponding to the afucosylated antibody (Mogamulizumab, Supplemental Fig. 2), indicating that even the non-glycosylated FcγRIIIa may exhibit sufficient affinity to an afucosylated antibody.

Thomann, *et al*., and Dashivets *et al*. reported that an affinity column with glycosylated FcγRIIIa separates fucosylated from non-fucosylated antibody^41,49^. On the other hand, non-glycosylated FcγRIIIa cannot distinguish fucosylated from non-fucosylated antibody in solution^40^. In our study, the non-glycosylated FcγRIIIa column does not exhibit significant resolution to fucosylated/non-fucosylated antibody (Mogamulizumab, Supplemental Fig. 2), supporting previous observations.

The column presented in our study was developed to increase stability, resulting in the fine resolution of fractions differing in their level of galatosylation. Based on our results, and the data from the group of Roche, it is conceivable that a mutated and glycosylated FcγRIIIa column may exhibit separation capability for both galactosylation and fucosylation simultaneously.

Our results showed that highly galactosylated antibody exhibits higher affinity to FcγRIIIa, and greater thermal stability, ADCC activity and CDC activity. These results highlighted the importance of controlling the heterogeneity of glycoform during preparation of therapeutic antibody to obtain consistent biological and therapeutic effects.

**Figure 5:**
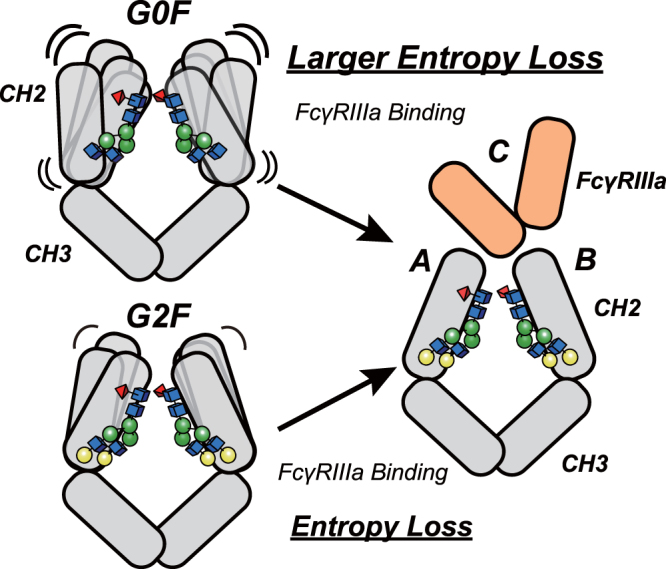
Binding model. Our data supports that the conformational entropy modulated by galactose governs the binding affinity of IgG to FcγRIIIa. The terminal galactose moiety stabilizes the hinge α-helical peptide located at the interface between the CH2 and CH3 domains. In the presence of N-glycans containing galactose, the CH2 domain remains in a more rigid conformation than that in the agalactosylated G0F glycoform. The rigid conformation yields a less unfavorable entropy change, facilitating the binding to FcγRIIIa. IgG-Fc and FcγRIIIa are represented in gray, and light orange respectively.

Crystallographic analysis revealed the detailed molecular interaction between Fc and non-glycosylated FcγRIIIa, although the structural data could not explain the molecular mechanism of how N-terminal galactose modulate the affinity to FcγRIIIa. Additional data was necessary to explain the role of galactose. Evidence from thermodynamic analysis and HDX-MS showed that not the static, but the dynamic behavior of CH2 is a key mechanism modulating the affinity of IgG-Fc for FcγRIIIa. Specifically, the galactose moiety decreased the conformational entropy of CH2, facilitating the engagement of Fc to FcγRIIIa (Fig. 5). Considering that the terminal α1, 6 galactose interacts with Lys246 of CH2^46,50^, our results support this interaction partially stabilizes CH2, resulting in enhanced affinity to FcγRIIIa.

In conclusion, our study further clarifies the mechanism of how glycosylation of Fc modulates the affinity to FcγRIIIa, and the impact of terminal galactose on biological function. This information will help guide better antibody therapy by rational molecular development of therapeutic antibody.

### Accession codes

The coordinates and structure factors for IgG-Fc in complex with Mut FcγRIIIa (non-glycosylated) have been deposited in the Protein Data Bank under accession code 5YC5.

## Electronic supplementary material

Supplemental Information
