# Supplementary material for: Assessing the Heterogeneity of the Fc-Glycan of a Therapeutic Antibody Using an engineered FcγReceptor IIIa-Immobilized Column
Source: Sci Rep. 2018 Mar 2;8:3955. doi: 10.1038/s41598-018-22199-8 (PMC5834517; doi:10.1038/s41598-018-22199-8)
Supplement: Supplementary file 1 — Supplemental Information [file 41598_2018_22199_MOESM1_ESM.pdf]

SUPPLEMENTAL INFORMATION

ASSESSING THE HETEROGENEITY OF THE FC-GLYCAN OF A THERAPEUTIC ANTIBODY USING AN ENGINEERED FCγRECEPTOR IIIA-IMMOBILIZED COLUMN.

Keywords: Method, Glycosylation, Effector Function, mAbs, FcγRIIIa column.

Masato Kiyoshi[1,*], Jose M.M. Caaveiro[2,3], Minoru Tada[1], Hiroko Tamura[2], Toru Tanaka[4], Yosuke Terao[4], Koldo Morante[2], Akira Harazono[1], Noritaka Hashii[1], Hiroko Shibata[1], Daisuke Kuroda[2], Satoru Nagatoishi[2], Seigo Oe[4], Teruhiko Ide[4], Kouhei Tsumoto[2,5,6], Akiko Ishii-Watabe[1].

[1]Division of Biological Chemistry and Biologicals, National Institute of Health Sciences, Tokyo, 158-8501, Japan, [2]Department of Bioengineering, School of Engineering, The University of Tokyo, Tokyo 113-8656, Japan, [3]Graduate School of Pharmaceutical Sciences, Kyushu University, Maidashi, Higashi-ku, Fukuoka, 812-8582, Japan, [4]Tosoh Corporation, Hayakawa, Ayase 252-1123, Japan. [5]Institute of Medical Sciences, The University of Tokyo, Shirokanedai, Minato-ku, Tokyo 108-8639, Japan, and [6]Laboratory of Pharmacokinetic Optimization, Center for Drug Design Research, National Institutes of Biomedical Innovation, Health and Nutrition, Ibaraki City, Osaka 567-0085, Japan.

*Correspondence and requests for materials should be addressed to M.K. (email: m.kiyoshi@nihs.go.jp)

(a)

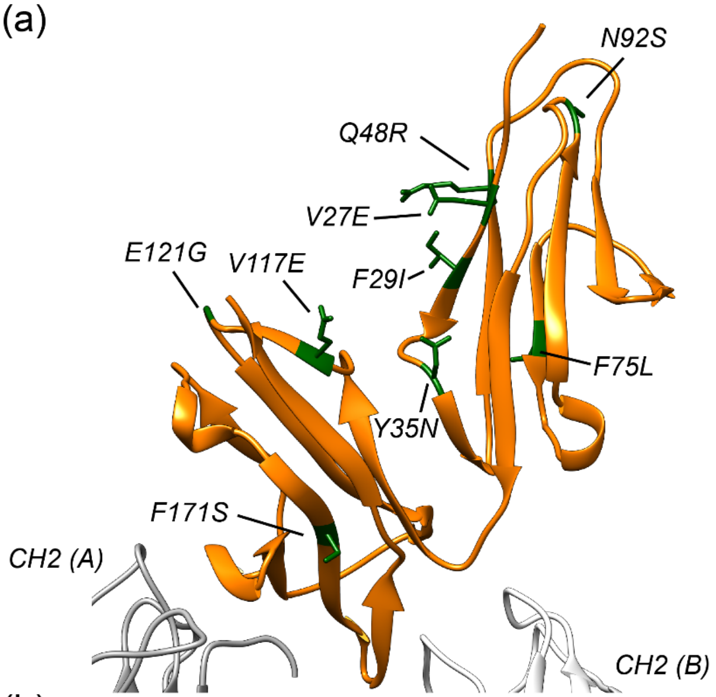

(b)

```
              20        30        40        50        60
FcgRIIIa Mut  RTEDLPKAEVILEPQWNRVLEKDSVTLKCRGAYSPEDNSTQWFHNESLIS
FcgRIIIa WT   RTEDLPKAVVFLEPQWYRVLEKDSVTLKCQGAYSPEDNSTQWFHNESLIS

              70        80        90        100       110
FcgRIIIa Mut  SQASSYLIDAATVDDSGEYRCQTSLSTLSDPVQLEVHIGWLLLQAPRWEF
FcgRIIIa WT   SQASSYFIDAATVDDSGEYRCQTNLSTLSDPVQLEVHIGWLLLQAPRWVF

              120       130       140       150       160
FcgRIIIa Mut  KEGDPIHLRCHSWKNTALHKVTYLQNGKGRKYFHHNSDFYIPKATLKDSG
FcgRIIIa WT   KEEDPIHLRCHSWKNTALHKVTYLQNGKGRKYFHHNSDFYIPKATLKDSG

              170       180       190
FcgRIIIa Mut  SYSCRGLVGSKNVSSETVNITITQG
FcgRIIIa WT   SYFCRGLVGSKNVSSETVNITITQG
```

Supplemental Figure 1. Mutated residues in FcγRIIIa.

(a) FcγRIIIa, chain A of CH2, chain B of CH2 are colored in orange, gray, and white, respectively. The mutated residues are depicted in green. (b) The sequence alignment of Mut FcγRIIIa of this study and that of wild type. The sequence number was adopted from the Uniprot database. The mutated residues are colored in green. Residues interacting with Fc are colored in magenta.

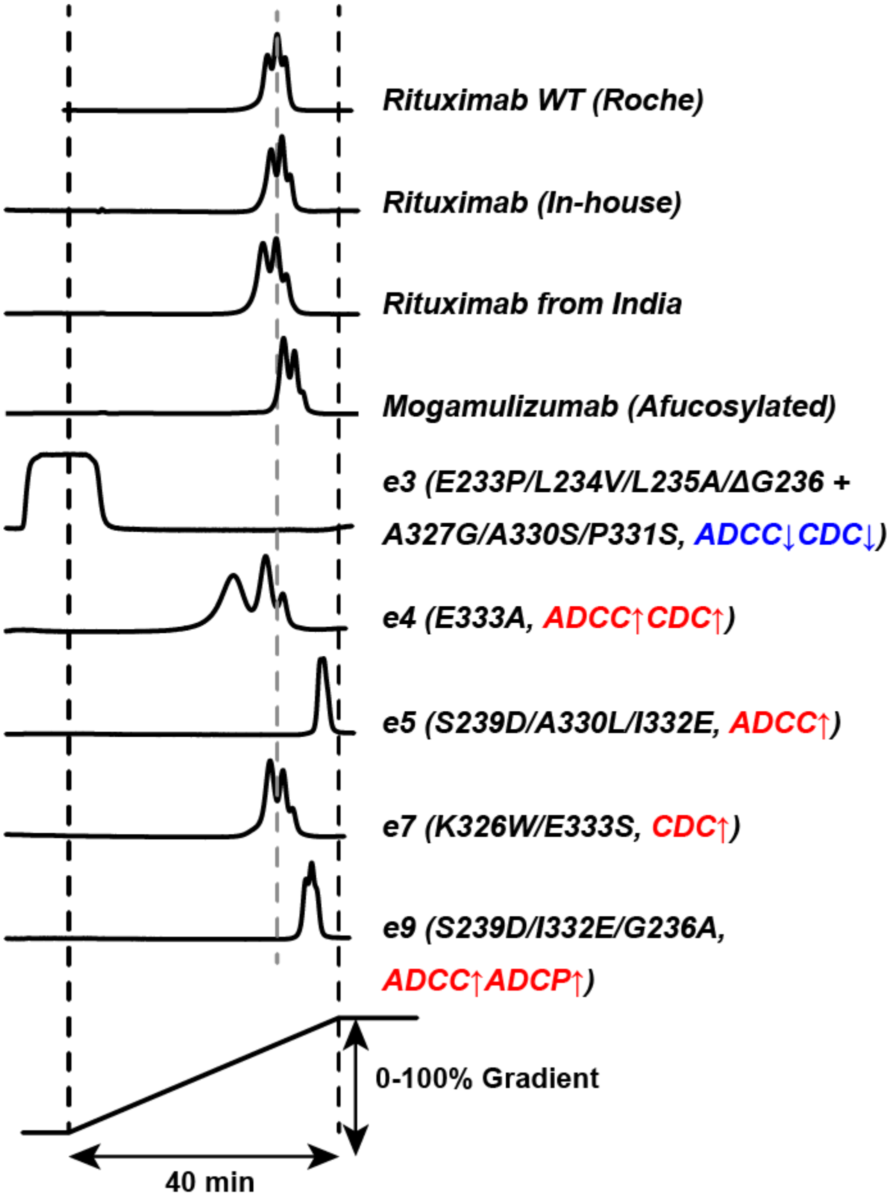

Supplemental Figure 2. Analytical chromatography using FcγRIIIa-immobilized column.
The mutation information was derived from InvivoGen website.

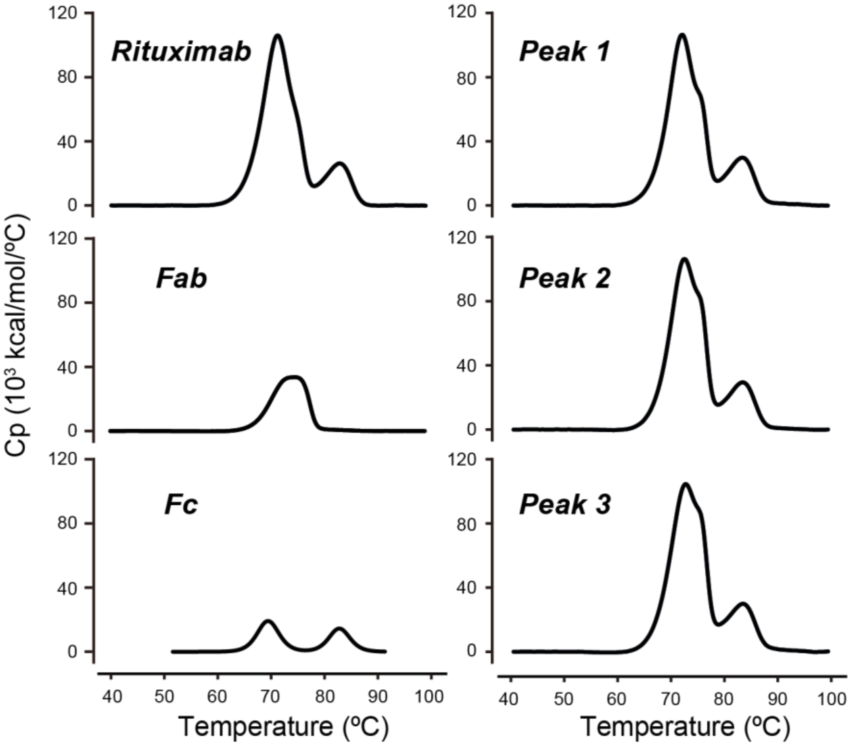

Supplemental Figure 3. DSC analysis of each IgG fraction. Thermal stability of each IgG glycovariant was determined using DSC. The thermograms of IgG before the chromatographic step is shown in the top-left. We observed differences in the partially overlapped peak at around 76 ºC next to the first transition.

Supplemental Table 1. Kinetic parameters of binding between antibody and WT FcγRIIIa, and Mut FcγRIIIa.

WT FcγRIIIa (glycosylated)			
**Protein**	k_{on} **(× 10^{4} M^{-1}s^{-1})**	k_{off} **(× 10^{-2} s^{-1})**	K_D **(nM)**
**Peak 1**	8.2 ± 0.04	1.5 ± 0.03	180
**Peak 2**	7.2 ± 0.04	9.3 ± 0.02	130
**Peak 3**	6.7 ± 0.03	6.4 ± 0.02	95

Mut FcγRIIIa (non-glycosylated)			
**Protein**	k_{on} **(× 10^{4} M^{-1}s^{-1})**	k_{off} **(× 10^{-2} s^{-1})**	K_D **(nM)**
**Peak 1**	5.2 ± 0.18	7.8 ± 0.23	1490
**Peak 2**	3.7 ± 0.10	3.8 ± 0.10	1010
**Peak 3**	9.0 ± 0.19	6.3 ± 0.14	701

Supplemental Table 2. Data collection and refinement statistics. Statistical values given in parenthesis refer to the highest resolution bin.

**Data Collection**	**Fc + Mut-FcγRIIIA (non-glycosylated)**
Space Group	P 2_1 2_1 2_1
Unit cell	
a, b, c (Å)	72.8, 101.4, 122.6
α, β, γ (°)	90.0, 90.0, 90.0
Resolution (Å)	46.9 – 2.71
Wavelength	1.000
Observations	151,026 (20,452)
Unique reflections	25,395 (3,324)
$R_{merge.}$	0.131 (0.848)
$R_{p.i.m.}$	0.058 (0.370)
Half-set correlation $CC_{1/2}$	0.995 (0.842)
$I / \sigma\ (I)$	9.6 (2.0)
Multiplicity	5.9 (6.2)
Completeness (%)	100 (100)
**Refinement Statistics**	
Resolution (Å)	46.9 – 2.71
R_{work} / R_{free} (%)	20.9 / 27.3
No. protein chains	3
No. atoms	
Protein	4,751
Carbohydrate	209
Other	1
Water	35
B-factor (Å^2)	
Protein	55.2
PPIX	61.9
Others	77.8
Water	40.0
Ramachandran Plot	
Preferred (%)	90.6
Allowed (%)	9.6
Outliers (%)	0
RMSD Bond (Å)	0.012
RMSD Angle (°)	1.67
PDB identification code	5YC5

Supplemental Table 3. Thermal stability of purified IgG evaluated by DSC.

**Protein**	T_{M1} **(°C)**	T_{M2} **(°C)**	T_{M3} **(°C)**	ΔH **(kcal/mol)**
**Rituximab**	71.6 ± 0.02	75.5 ± 0.06	83.1 ± 0.04	9.8×10^2
**Peak 1**	72.1 ± 0.02	75.8 ± 0.03	83.2 ± 0.03	9.8×10^2
**Peak 2**	72.5 ± 0.03	75.7 ± 0.03	83.3 ± 0.03	9.9×10^2
**Peak 3**	72.6 ± 0.04	75.7 ± 0.03	83.4 ± 0.04	10.0×10^2
